# Current Research and Challenges in Bitumen Emulsion Manufacturing and Its Properties

**DOI:** 10.3390/ma15062026

**Published:** 2022-03-09

**Authors:** Ahmed Al-Mohammedawi, Konrad Mollenhauer

**Affiliations:** Engineering and Maintenance of Road Infrastructure, Transportation Institute, University of Kassel, Mönchebergstraße 7, 34125 Kassel, Germany; k.mollenhauer@uni-kassel.de

**Keywords:** bitumen emulsion, formulation, emulsification temperature

## Abstract

The global increase of road infrastructure and its impact on the environment requires serious attention to develop sustainable and environmentally friendly road materials. One group of those materials is produced by using bitumen emulsion. However, there are still scientific and technical obstacles standing against its regular application. The bitumen emulsion formulation process and compositional optimization are subjected to a high number of degrees of freedom. Consequently, obtaining the desired product is mostly based on a series of random and tedious trials because of the enormous number of tests that are carried out to meet the required properties, such as emulsion stability, viscosity, droplet size (and distribution), and bitumen emulsion chemistry. Several pre-established formulation procedures have been presented in the literature. Some of them have technical limitations to be utilized for practical industrial application, whereas others are still not understood enough to be applied in bitumen emulsion formulation. Therefore, discussing some important issues in this field could be useful to offer a practical guide for bitumen emulsion manufacturers when trying to formulate a well-defined bitumen emulsion to best fit its use in pavement infrastructure rather than to simply to meet standard specifications. This review paper aims to enable the ultimate potential of bitumen emulsion by further reviewing the research progress of bitumen emulsion manufacturing and discussing the literature available up to now on this topic, in the realm of bitumen emulsion manufacturing and emulsion chemistry.

## 1. Introduction

Bitumen emulsion is becoming more popular in the pavement construction sector. its applications are quite diverse, including cold recycling mixtures, tack coating, and surface treatment. Bitumen emulsions are an innovative method for liquifying bitumen by dispersing it in water. Bitumen emulsions have a significant advantage over hot bitumen since their operations need less energy because their viscosity is lower than that of hot bitumen. Furthermore, there are fewer hazards of fire and burns, and the process consumes less energy [1]. As a result, the cold mix emits less ozone-depleting hydrocarbons [2]. As an emulsion, bitumen emulsion is considered as an oil in water (O/W) emulsion system with water (or soap) as the continuous phase and bitumen as the dispersed phase. For phase compatibility, non-ionic, anionic, or cationic surfactants are added at a concentration of 1–2 weight percent of the total bitumen emulsion mass. According to industrial experience [3], the proper emulsion for road applications has a high bitumen content, which normally ranges from 60 to 70%, with a unimodal droplet size distribution and an average droplet size ranging from 5 to 15 μm. Surfactants lower the viscosity of the system in comparison to the initial viscosity of the bitumen. The amphiphile molecule (surfactant) prevents droplet coalescence by decreasing the interfacial tension between bitumen and water, thereby stabilizing the system and allowing emulsification [4]. Bitumen emulsion’s main properties, including type, average drop size, size distribution, rheology, and stability, as well as its further use properties, such as adhesion on the substrate, must be tailored according to the pavement application (i.e., cold recycling, chip sealing).

Cold mixture technology presents a new set of challenges to engineers who are traditionally used to work with hot mixes. Whereas hot mixtures rely on the viscoelastic properties of bitumen, emulsion mixtures introduce a new series of conditions that must be controlled in order to successfully produce and lay these materials. One of the main obstacles that stands against the vast usage of bitumen emulsion in cold recycling is the low early mechanical performance, such as the low bearing capacity and adhesion. More precisely, it is the poor ability of bitumen emulsion to develop sufficient bonding in the mixture skeleton, which normally takes time and is generally caused by the poor compatibility between bitumen emulsion and aggregate (even more complex with RA aggregate due to aged bitumen). The surface chemistry of the aggregate begins to have an important role, and emulsions must be tailored to the mineralogy of different aggregate types. The challenge of emulsion formulation is generally manipulated by selecting the appropriate components (surfactant, bitumen, aqueous phase) and their quantities. The optimized formulation should provide an emulsion with the desired emulsion breaking time, either during mixing or compaction, and proper aggregate coating, after which there is an increase in strength over time. However, the characteristics of initial workability or being able to stockpile the material and the subsequent development of mechanical strength in situ form conflicting requirements. The optimization of the formulation and emulsification technique may be made reasonably straightforward by segregating and treating the different factors independently [5]. Bituminous emulsions have been subjected to comparably little scientific study and even fewer publications in the area of formulation techniques and chemistry.

**Figure 1:**
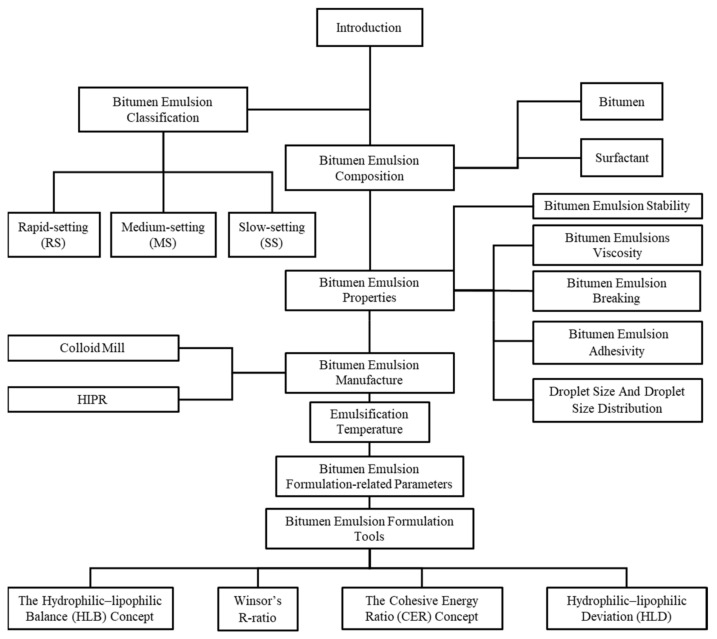
The systematic framework of discussion in this review.

This review paper, therefore, aims at consolidating and examining the literature available on this subject, which is relatively extensively scattered across the literature and patents in various research fields, in order to further ultimate the application of cold bitumen technology in practice. Figure 1 illustrates the framework of discussion in this review.

## 2. Bitumen Emulsion Composition and Classification

### 2.1. Overview

Bitumen emulsions are composed of water, surfactant, and bitumen itself. The emulsion is formed in two phases, using two immiscible liquids. Thanks to the electrostatic charges induced by the surfactant, the emulsion particles are suspended in the aqueous phase. Within this concept, surfactants can be defined as emulsifying agents that are active on the surface of emulsified substances; thus, they are known as surfactants (surface active agents). Usually, 50–75% of the emulsion is bitumen; therefore, it is considered the most important component of bitumen emulsion [4].

### 2.2. Bitumen

Bitumen is known as a complex material that results from crude oil distillation. Bitumen is used in various construction sectors, mostly as a binder in asphalt pavement [4,6,7,8,9]. Bitumen can be described as a mixture of four broad chemical groups: resins, asphaltenes, aromatics, and saturates. The first two groups, due to their basic and acid functional groups, are considered the most polar compounds, and therefore, they affect the interfacial properties affected [10]. The mechanism behind that is that these polar compounds can migrate and adhere to the water when bitumen droplets are emulsified in water. In this way, they can act as natural surfactants [11]. Other components, such as wax crystals and naphthenic acids, can also act as natural surfactants and can affect the interfacial property of the bitumen water system, but only in the case of low or high pH aqueous phases [12,13,14,15,16]. Thus, bitumen type and source are paramount parameters in the chemical interaction of the surfactants with the bitumen in producing the emulsions.

### 2.3. Surfactant

A surfactant’s basic function is to reduce the surface tension of an emulsion and prevent the coalescence of the droplets. Besides, they control bitumen emulsion characteristics for instance stability, viscosity, breaking, and adhesivity. The latter is very important in the strength development of a cold recycled mixture [4,17]. The majority of surfactants have been utilized in the emulsification of bitumen. Anionic surfactants were the most common in the beginning, but cationic surfactants have gradually taken control since the 1950s [18].

**Figure 2:**
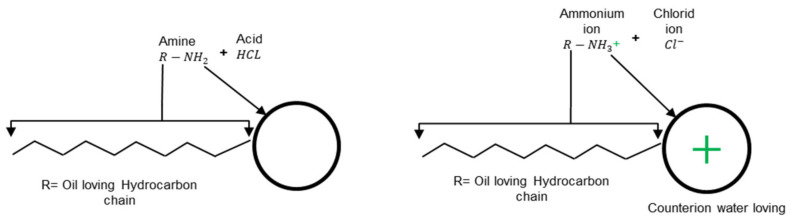
Example of a cationic soap production adapted from [5].

A surfactant molecule consists of two parts, namely the polar (hydrophilic head group) and the non-polar (hydrophobic chain group). The first makes it water-soluble, and this group is particularly crucial for the aqueous surfactant solution characteristics [19]. Bitumen emulsions are categorized as anionic, cationic, non-ionic, or zwitterionic based on the charge carried by the head group [20]. In general, cationic surfactants are fatty amines such as imidazolines, amidoamines, and diamines [21] that can be converted into soap by mixing them with a suitable acid, normally HCl, as shown in Figure 2. It can be seen that the outcomes from this reaction are ammonium compounds that have nitrogen (N) atoms with a positive charge in their head group ($R-NH_{3}^{+}Cl^{-}$). Electrovalent and polar head groups have a positive charge, and this charge is migrated to the bitumen droplet surface.

**Figure 3:**
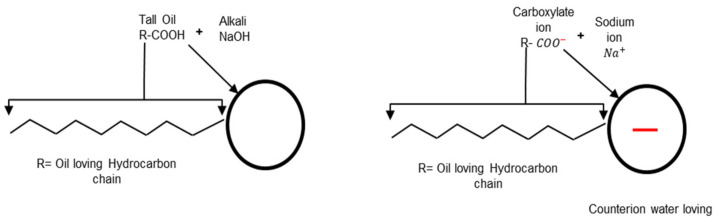
Example of an anionic soap production adapted from [5].

Anionic surfactants are fatty acids such as lignin, tall oil, and rosin extracted from trees [22,23]. Again, they are converted to soap by reaction with an alkali, usually, sodium hydroxide (NaOH), as shown in Figure 3. The result is the carboxylate compounds that have negatively-charged oxygen (O) atoms in their head group ($R-COO^{-}Na^{+}$). Electrovalent and polar head groups are negatively charged, and their positive charge is migrated to the surface of the bitumen droplet. In addition, fatty quaternary ammonium salts are another type of emulsifier used to make cationic emulsions. These types of surfactants are suitable and stable cationic surfactants because they are water-soluble salts and require no acid addition [21,24,25].

### 2.4. Bitumen Emulsion Classification

Emulsions can also be categorized by the rate at which droplets of bitumen coalesce and return to bitumen. This is related to the rate at which the emulsion becomes unstable and breaks after mixing with aggregate and/or filler. Bitumen emulsions are divided into Rapid-Setting (RS), M-Setting (MS), and Slow-Setting (SS). In addition, bitumen emulsions are classified based on EN 13808 [26], with various letters and numbers indicating viscosity and basic bitumen type. Table 1 shows the abbreviations for cationic emulsions [27]. For example, C69BF3 70/100 is a cationic bitumen emulsion made from 70/100 pen-grade bitumen with a 69% bitumen fraction that contains more than 3% (m/m) flux and a breaking class of 3.

## 3. Bitumen Emulsion Properties

The most essential performance requirements of bitumen emulsions are stability, viscosity, breaking, adhesion, droplet size, and dispersion. The perfect emulsion is stable under the conditions of storage, transport, and application, but it should return to bitumen quickly after application, leaving a binder with the properties of the original bitumen, firmly adhering to the aggregate, and consequently providing enough bearing capacity for a timely construction process [4].

### 3.1. Bitumen Emulsion Stability

**Figure 4:**
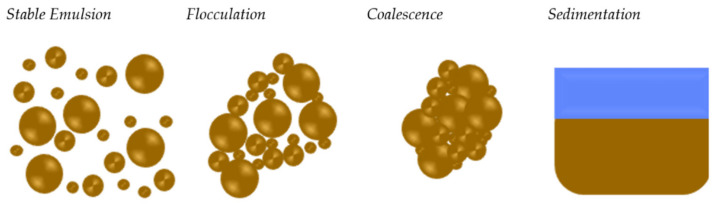
Bitumen emulsion flocculation, coalescence, and sedimentation adapted from [5].

Emulsion stability is an important parameter to consider and monitor when producing a bitumen emulsion. The tendency of an emulsion to alter characteristics over time is referred to as its emulsion stability [28]. In general, it is controlled by the interactions between the surfactant and the water/bitumen interfaces. During the storage, bitumen droplets begin to approach one another due to low surfactant concentration at the interfaces; therefore, contact between the two droplets is expected, and what is called the flocculation phenomenon occurs [29]. When there is no mechanical agitation in the system, this is what happens during emulsion storage. If steps are not taken to reverse flocculation, coalescence can occur (see Figure 4). With coalescence, the surfactant layer between the droplets is compromised, allowing the droplets to contact one another. During this process, water can become trapped within the new larger droplet. Since there is no longer a physical barrier between the emulsion droplets, the droplets cannot be separated [30]. This last mechanism has substantial dependence on formulation [31] in terms of the bitumen grade, bitumen temperature, soap temperature, and surfactant type [32,33,34]. During storage, bitumen emulsion is subjected to gravitational force, leading to the bitumen droplets being dragged down to the bottom due to the difference in densities of the disperse and the continuous phases forming the sedimentation phenomenon.

### 3.2. Bitumen Emulsions Viscosity

Emulsion viscosity is an important performance characteristic of bitumen emulsions. In cold recycled mixes, low viscous emulsions may drain off the aggregate or affect the aggregate/binder adhesion evolution, which in turn affects the gaining of the mechanical strength. On the other hand, in chip seal, low viscosity emulsions are likely to run off the road, whereas highly viscous emulsions may not distribute well over the surface. In this manner, bitumen emulsion viscosity affects the performance of the final product. Bitumen emulsion viscosity is affected by factors such as emulsion particle size and particle size distribution [35], the bitumen to water ratio [36], the surfactant type [37], and the presence of salt in the bitumen [38], which can lead to a higher viscosity during storage of the emulsion [34].

However, it is a challenging task to regulate these variables autonomously during emulsion manufacturing. This is because certain characteristics, for instance surfactant concentration and interfacial tension, are dependent parameters. For example, any change in the interfacial tension will change the droplet’s size (and its distribution), which in turn changes the bitumen emulsion viscosity. Usually, the emulsion viscosity raises as the average droplet size decreases, while the emulsion viscosity drops down as the droplet size distribution becomes narrower [39,40,41]. Besides, the emulsion viscosity can also be affected by the surfactant content, as it can be increased when the surfactant content is increased due to the droplet’s size reduction. In contrast, when the viscosity goes down, the system will experience an ultra-low interfacial tension [42]. This system is completely near to the ideal formulation, which will be discussed in detail in Section 7.5, and droplet size is often quite tiny. Nevertheless, these systems are exceedingly unstable; for example, droplet coalescence happens very quickly, even directly after emulsification. Besides, the emulsion viscosity can also be affected by the surfactant chemical structure. Furthermore, some surfactants have a unique effect, as they can form multiple emulsions (W/O/W). Therefore, the emulsion viscosity is very important to be controlled as it affects the product during the manufacturing, storage, and mechanical performance during the service [38,40,43].

### 3.3. Bitumen Emulsion Breaking

Bitumen emulsion is intended to break when it comes into contact with aggregates, leaving a binder layer on and between the aggregate particles. The overall process, also known as emulsion settling, is a complicated phenomenon that determines the ultimate performance of the pavement. Emulsion breaking is not comprehensively understood, and there are numerous approaches of explanation for related phenomena. Various processes occur at the same time during the breaking. On this basis, several research studies on bitumen emulsion breaking mechanisms have been conducted, focusing primarily on the surfactant, the kind and amount of additive, and the bitumen emulsion preparation conditions. According to Gorman et al., the most critical parameters influencing the breaking process of bitumen emulsion are bitumen emulsion characteristics and aggregate surface properties [44]. According to Hagen et al., the surface properties of the aggregate have a significant impact on the breaking speed and the adhesion of the bitumen emulsion [45]. Lyklema [46] and Castillo [47] believe that surfactant desorption on the bitumen surface and adsorption on the aggregate surface influence bitumen emulsion breaking. Read and Whiteoak revealed that the higher the temperature is, the higher the breaking speed [4]. In general, breaking is regulated by physiochemical interactions [48]. Marchal et al. noticed that the quantity of surfactant that is not attached to bitumen droplets or adsorbed on the surface of the aggregate has a significant impact on the bitumen emulsion’s breaking speed [49,50]. Therefore, it essentially involves different breaking mechanisms such as the surfactant adsorption breaking mechanism, the water evaporation breaking mechanism, and the pH change breaking mechanism [5].

#### 3.3.1. Surfactant Adsorption Breaking Mechanism

**Figure 5:**
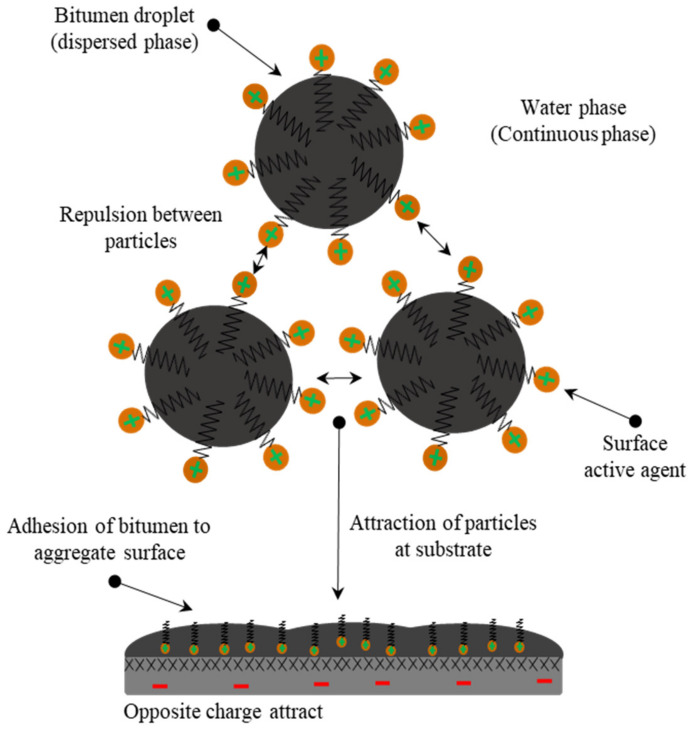
Schema of bitumen emulsion droplet changes adapted from [4].

Molecules of the surfactant are disseminated among bitumen globules and suspended in water, with micelles being formed by some of their ions, but in stable conditions, the emulsion is in equilibrium (Figure 5) until the adsorption of these ions by polarized mineral aggregates. Consequently, the emulsion breaks at a rate depending on surfactant adsorption in mineral aggregate, affected by the chemical properties of the latter and the surface area [49,50]. A schema of the breaking mechanism due to adsorption is shown in Figure 5. Poirier et al. [51] examined the adsorption of a cationic surfactant at the bitumen/water interface in order to estimate the stability of bitumen emulsions. The adsorbed quantity of the surfactant was tracked in this investigation by varying concentrations in the bulk aqueous phase. The results revealed that, at the surfactant’s CMC, the adsorption isotherm exhibited a plateau, but no multilayer adsorption occurred. Furthermore, it was found that there is a strong correlation between the adsorbed amount and the electrokinetic potential variations [51].

#### 3.3.2. Water Evaporation Breaking Mechanism

Water evaporation from the emulsion, affected by environmental conditions including wind speed, humidity, and temperature, has major impacts on breaking. Evaporation causes the concentration of bitumen droplets in the emulsion to increase, thus increasing the inter-droplet contact and relative homogenization and diffusing surfactants into the solution from the interfaces to form surfactant micelles, due to decreasing the interfacial area. In industrial applications (e.g., cement and asphalt pavement construction), water is characteristically trapped within structures; similarly, in bitumen, a significant quantity of water is sealed in closed pores, and evaporative processes can go on for years, which is why CRM takes many years to reach full strength. The evaporative breaking process is represented in Figure 5 [52].

#### 3.3.3. pH Change Breaking Mechanism

Acidic or alkaline reactions cause surfactants to react, forming surfactant soaps, due to surfactants’ surface charges being dependent on pH, as discussed previously. For instance, cationic surfactants such as fatty amines are soluble in acid but are insoluble in water. Fatty amines are dissolved by HCl to produce water-soluble soaps, which can then be blended in the colloid mill with hot bitumen to yield bitumen emulsion. Surfactants’ functions can therefore be compromised when cement or mineral aggregate engage with cationic emulsions, due to increased pH; cement is particularly potent in affecting the pH of cationic emulsion. Consequently, the setting time and the stability of bitumen emulsions are normally gauged concerning active filler [53]. It should be noted in this regard that some surfactants, including quaternary amines, retain their functionality even when pH changes [5].

### 3.4. Bitumen Emulsion Adhesivity

**Figure 6:**
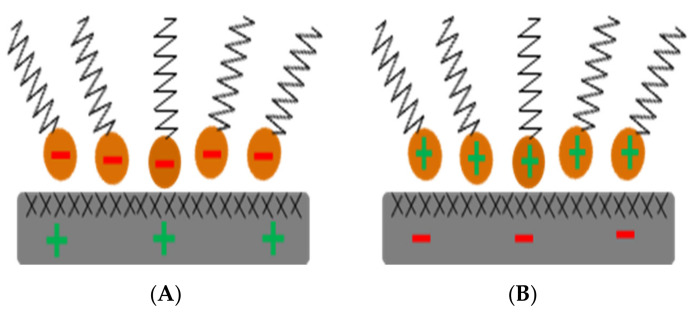
Surfactant adsorption by ionic change: (**A**) anionic; (**B**) cationic adapted from [4].

Considering the right type of aggregate and bitumen emulsion is very important to characterize the compatibility of CRM in terms of adhesion performance and water sensitivity. Therefore, the choice of the surfactant with the type of mineral material can be optimized to achieve the maximum efficiency of preparation and use of the bitumen emulsion. However, the research on the surface characteristics of the aggregate and bitumen emulsion chemistry is rarely reported. Meanwhile, each aggregate-bitumen emulsion combination has a distinct chemical composition, which influences the connection between the aggregate and the bitumen [44,54,55] and therefore affects the CRM mechanical performance (i.e., bearing capacity and water damage sensitivity) [56,57,58]. In the case of cationic emulsions, the use of an aggregate with a surface charge similar to that of bitumen, such as limestone, results in poor adhesion between the bitumen and the aggregate, as well as a delay in the curing time [59,60]. This phenomenon, as shown in Figure 6, is governed by the physiochemical adsorption of the bitumen and surfactant onto the mineral filler/and or aggregate surface, which is in turn controlled by several factors, including Van der Waals attraction forces, electrostatic forces, or covalent and electrovalent bindings, and the film adhesion. The formation of adhesion film depends on the bitumen and aggregate type. In an anionic emulsion, bitumen droplets are surrounded by a negative charge. If the anionic bitumen emulsion is mixed with an acid-nature aggregate (i.e., silica), then emulsion could be destabilized rapidly and therefore cause early adhesion. Recently, a study on molecular dynamic simulation and conductivity experiments was conducted to systematically explore the adsorption of different anionic surfactants on calcium carbonate and silica. Results indicated that the adsorption of the anionic surfactants on the SiO_2_ surface was stronger than that on the CaCO_3_ surface [61].

When cationic bitumen emulsion is combined with a basic aggregate, however, a possible interaction between the surfactant and the carbonate anion might occur. Adsorption of the produced salt by the solid surface improves adhesion. Hu et al. [62] carried out a study on cement bitumen emulsion mortar to investigate the adsorption of different bitumen emulsions on a cement grain surface by the means of characterizing the particle size variation using a laser particle size analyzer. It was found that bitumen droplets in cationic bitumen emulsion were much more likely to be adsorbed to cement grains in comparison with those in anionic bitumen emulsion [62]. These chemical reactions can explain why cationic bitumen emulsions are preferred in cold mixes. In addition, the bitumen adsorption on the substrate can also be optimized by lowering the surfactant concentration slightly below the critical micelle concentration (CMC). Meanwhile, a surfactant concentration higher than the CMC is necessary to increase the stability of the unstable thermodynamic emulsion [63]. The critical micelle concentration (CMC) is an important characteristic that can sometimes be forgotten or left out of the discussion during the emulsification process; it is for the surfactant concentration where adding any more surfactants would cause micelles to form. It depends on the surfactant, medium (usually water), temperature, pressure, and salinity.

Al-Mohammedawi and Mollenhauer [53] conducted a study on the effect of the chemical nature of fillers (both acidic and basic fillers) on the mechanical properties of cationic bitumen emulsion mastic. It was found that the filler with the basic nature shows high reactivity in terms of pH value, which is reflected in the fatigue and rheological performance that was believed to be due to the high filler/bitumen emulsion compatibility [53,64]. However, this study needs to be supported by microscopic and microstructure analysis to obtain more information about the surfactant and bitumen adsorption on filler particles. In this regard, there is a growing research interest in employing the enhanced mesoscopic methods that can be used efficiently to identify the colloidal forces between the bitumen droplets in the aqueous phase [65,66,67] and between bitumen and aggregate [68,69,70,71,72,73]. Knowing that these techniques are normally applied for Athabasca bitumen, they can be adapted to be used in bitumen emulsion research area, as both fields share the same underlying physical chemistry [10,14,74,75,76,77,78]. In this way, several investigations have been conducted to investigate the surfactant/bitumen compatibility using different techniques such as surface free energy, fluorescence spectroscopy, neutron scattering, interfacial viscoelasticity, and Raman spectroscopy [79,80,81,82,83,84,85]. Jin et al. [86] investigated the synthesis of a composite surfactant using an OP-10 cationic surfactant [86]. The impacts of the manufacture parameters and formulation on the surfactant emulsification properties, service performance, and surface activity were investigated. Tan et al. [87] that surfactants have a considerable retarding impact on cement hydration, which is related to the kinds of surfactants and their doses [87]. As a result, a suitable surfactant with a low retarding impact on active filler and its appropriate dose is advised. Consequently, when bitumen emulsions are employed in cold asphalt mixtures, the chemical characteristics of surfactants and aggregates should be considered throughout the design process to achieve the desired adhesive property [88,89,90].

### 3.5. Droplet Size and Droplet Size Distribution

**Figure 7:**
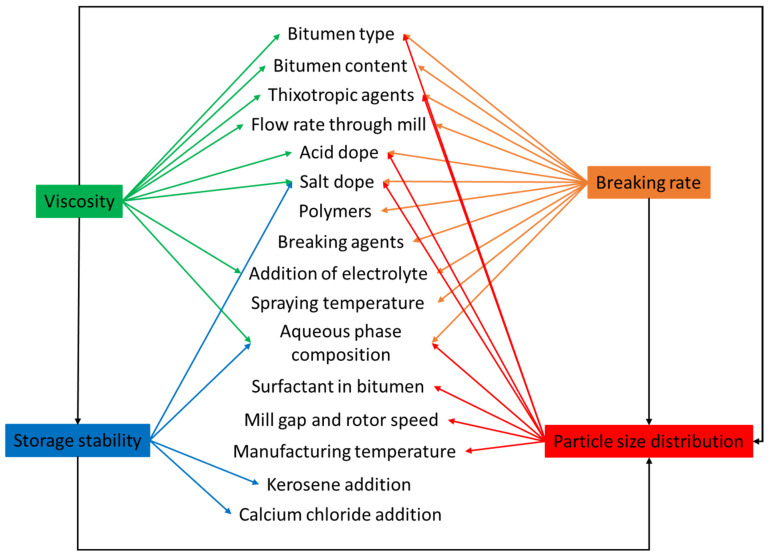
The interrelationship between manufacturing variables and properties for bitumen emulsion adapted from [103].

The granulometry of an emulsion can be characterized as a function of mass, volume, and surface of droplets. Nonetheless, the mean average size is the most commonly used definition. Emulsions are classified into two types based on droplet size: macro-emulsions and micro-emulsions. Droplet sizes in macro-emulsions are in the size range of micrometers (μm), whereas droplet sizes in micro-emulsions are in the size range of nanometers (nm). Microemulsions are thermodynamically stable, which means they have high stability due to their small droplet size, but macroemulsions are thermodynamically unstable (the larger the droplet size, the lower the stability) [91,92,93]. The size of the droplet is determined by several variables, as shown in Figure 7. These variables are also significant in emulsion stability [94]. During the emulsion manufacturing process, bitumen emulsion with various droplet sizes is produced. This is due to mechanical factors such as interface shearing, gap width, and so on, which results in polydispersity [95,96]. Increasing the emulsification time typically results in smaller droplets and narrower distribution [97]. The speed of emulsification has a comparable influence on the droplet size and its distribution. Bitumen emulsion particles are typically 0.1–20 μm in size [5,98]. Not only does droplet size affect stability, but it can also change the emulsion’s rheological characteristics [19,91,99]. Moreover, the content of the surfactant has a significant impact on the size of the droplets. Smaller droplets are formed when the surfactant dosage rises. This may be explained by an increase in the ability to cover a larger interfacial surface as well as a quicker surfactant migration kinetic to the bitumen-water interface. According to Baumgardner, the particle size of bitumen emulsion may be varied by the formulation, raw ingredients (i.e., surfactant), and manufacturing equipment [98]. In this regard, Liu et al. pointed out that surfactants with increased surface activity resulted in emulsions with reduced average particle size diameters. They concluded that increasing the surfactant dose lowered the average particle size diameter [100]. These observations were later verified by [101]. Similarly, Gingras et al. indicated that when mill rotor speed augmented and emulsification temperatures dropped down [3], the average particle size decreased. The findings of [102] corroborated this notion. Gingras et al. investigated bitumen content and its impact on particle size. As the bitumen content reduced, they observed a reduction in droplet size. They ascribed this interaction to particles colliding more frequently when the bitumen content was higher [3]. Gutierrez et al., on the other hand, had a different observation. They examined a higher content of bitumen than what Gingras did, but their observations did not coincide [102]. More studies might lead to a more complete explanation; however, it is apparent that the bitumen content influences the particle size of the resultant emulsion. The preceding sections offered an overview of the variables that determine bitumen emulsion characteristics. However, it is nearly impossible to change one emulsion characteristic without affecting the others. Figure 7 illustrates this interrelationship.

## 4. Bitumen Emulsion Manufacture

**Figure 8:**
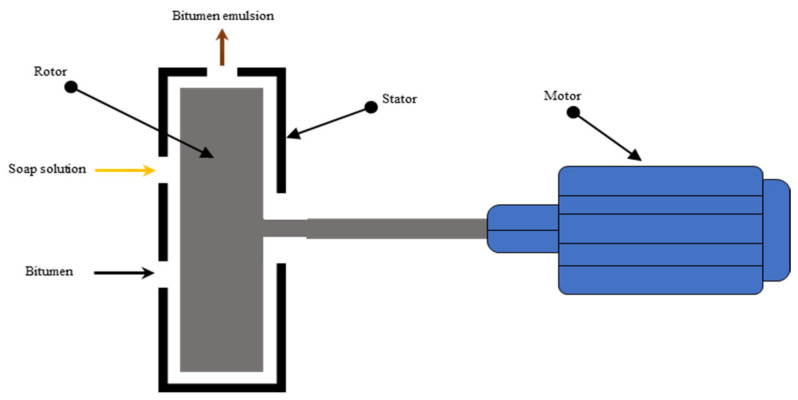
Colloid mill cross section adapted from [110].

**Figure 9:**
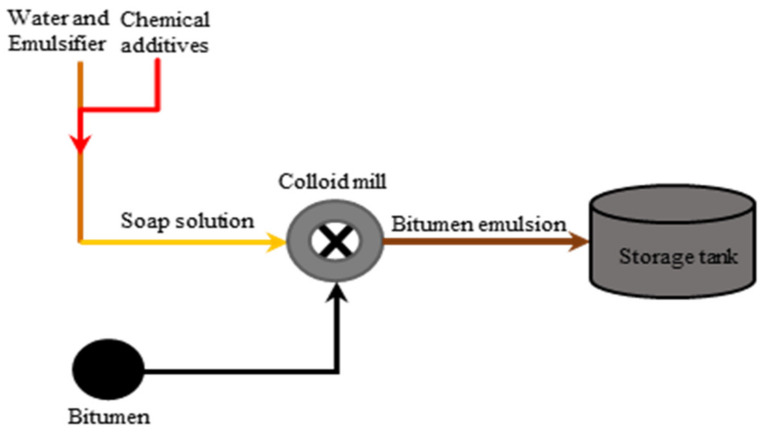
Schematic of a bitumen emulsion plant adapted from [4].

The production of bitumen emulsion is a complex process. In the industry, formulators must consider several aspects linked not only to the production process but also to the end attributes for which the emulsion has been developed. Mechanical parameters such as the emulsification method and its variables must be managed during the production of a bitumen emulsion [3,104,105]. There are two methods used to fabricate bitumen emulsions, namely colloid mill and High Internal Phase Ratio (HIPR). Colloid mill is the most commonly used equipment for emulsion manufacturing. Colloid mill, as illustrated in Figure 8, consists of a fast rotor (1000–6000 revs/min) in a stator; thanks to the generated shear forces, the bitumen is torn and stretched into globules, which are coated by surfactant and thus electrically charged. The mill’s geometric characteristics are instrumental in the distribution of particle sizes, with a common adjustable clearance range of 0.25 to 0.5 mm between the rotor and stator [106]. The colloid mill receives, firstly, surfactant mixed with water with temperatures ranging from 40–65 °C. Afterward, hot bitumen at 120–180 °C, with a viscosity of about 0.2 Pa s, which is suitable for emulsification, is then provided in the colloid mill gradually (see Figure 9). In some cases, emulsions may be thermally unstable or short-lived (i.e., stiffer bitumen or polymer-modified). During the emulsification process, the water phase compensates for the temperature to be below 90 °C, improving thermal stability [107,108]. For stiffer bitumen, higher temperatures should be provided, and more mill power should be used to generate bitumen droplets of the required size. However, the boiling point should be avoided [4]. For cold recycling purposes, emulsification generally occurs within a bitumen content of 60 to 70%. Above this range, the fabrication is inefficient due to the formation of very large drop particles, and emulsions have little storage stability. Furthermore, the droplet size achieved by this approach ranges from 5 to 10 µm; tiny droplet sizes are quite difficult to generate [109,110].

**Figure 10:**
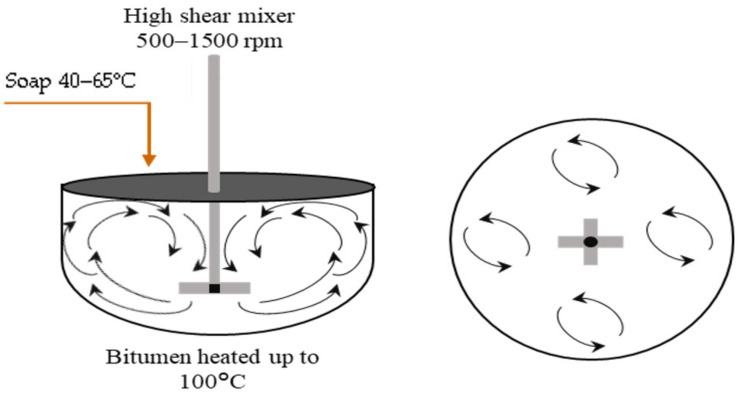
Schematic of bitumen emulsion manufacturing using the HIPR technique [113].

**Figure 11:**
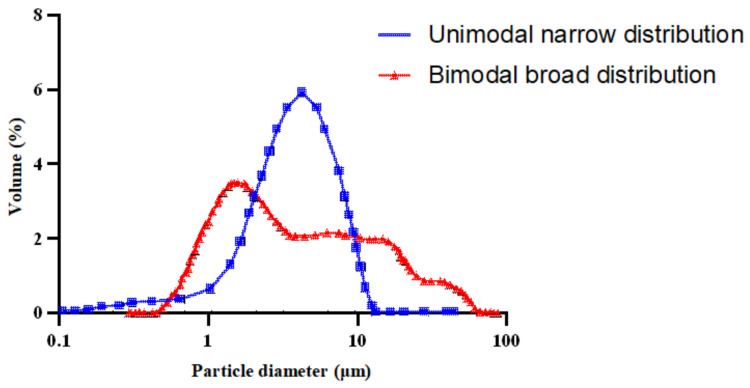
Unimodal and bimodal bitumen emulsion particle size distribution adapted from [117].

For smaller droplet sizes, another technique called High Internal Phase Ratio (HIPR) was introduced by Lissant et al. [111,112]. Within this technique, Samanos performed research on the manufacturing of bitumen emulsions for use in asphalt pavement manufacturing and maintenance, intending to achieve controlled emulsion properties through the employment of a dispersion phase in a suitable mixer. Based on this study, a lot of the studies were developed later [43,113] trying to optimize the manufacturing parameters (temperature of bitumen and soap, shear rate, material composition, and bitumen basic properties) [43,95,114]. The HIPR approach (Figure 10) employs phase inversion, which considers the physicochemical characteristics and the concentration of each component during fabrication. As a result, the type of emulsion produced, as well as its characteristics, may be tailored. This process entails a direct blend of a very viscous phase (1–5000 Pa s) with a second phase that is immiscible with the first and contains at least one surfactant. It produces a viscoelastic paste that may be diluted to the needed concentration of the dispersed phase using low shear (500–1500 rpm) and laminar flow. This process normally takes a short time. This technique may produce monomodal emulsions with extremely narrow particle size distributions and small mean droplet sizes of around 1 μm. Furthermore, this technique is capable of producing concentrated and extremely concentrated bitumen emulsions (70–95%) [102,115]. The droplet size of the emulsions generated by this method may be easily modified by varying the rotating speed, formulation parameters, or the concentration of the dispersed phase utilized during manufacturing. Unimodal emulsions have a single particle size distribution (produced within this method), but bimodal emulsions have two controllable droplet sizes and distributions, as illustrated in Figure 11 [40,116,117,118]. 

## 5. Emulsification Temperature

**Figure 12:**
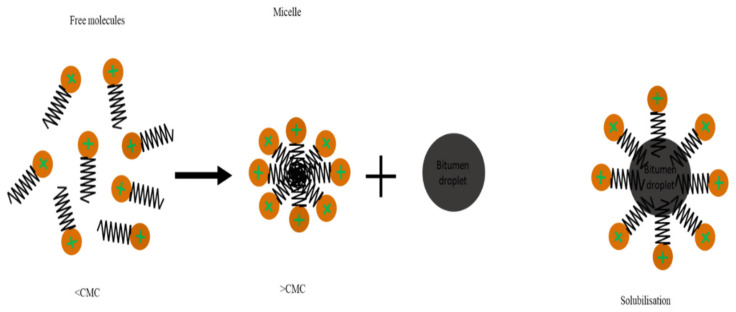
Schematics of micelle formation at concentrations above the CMC and bitumen droplet dispersion by micelle in the aqueous phase.

**Figure 13:**
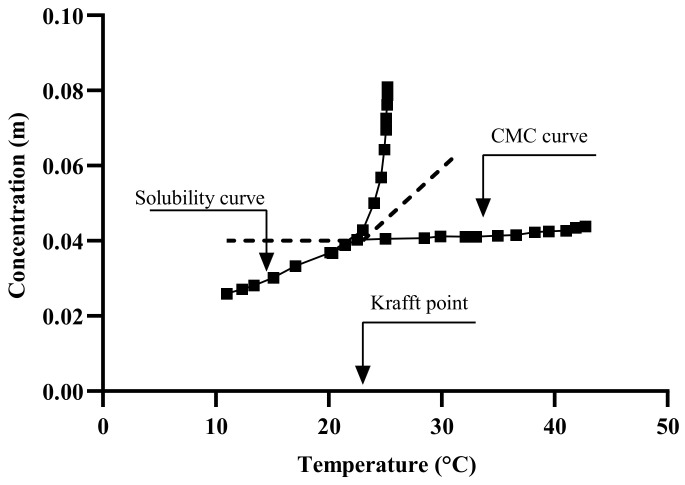
Conceptual chart of the Krafft point and CMC adapted from [131].

The emulsification temperature includes soap and bitumen temperatures, as well as the resulted emulsion temperature. Playing around with these temperatures could result in a bitumen emulsion with different properties. Therefore, these temperatures should be carefully defined and optimized along with the bitumen emulsion properties. This is significant because the boiling point can be reached during the emulsification process (this can be avoided by selecting the right soap/bitumen temperatures). The soap temperature has a significant impact on the solubility of ionic surfactants in water, as the solubility upsurges with increasing temperature until it reaches a limit value known as the Krafft Point Temperature (KT). Below the Krafft temperature, surfactant micelles cannot be formed as shown in Figure 12 and Figure 13 [119,120], and the surfactant is insoluble [121,122]. However, at the Krafft temperature, the surfactant’s solubility increases dramatically, and an additional temperature increment allows for micelle formation upon reaching the CMC [123]. This means that when temperatures rise, the hydrophilic character of the ionic surfactants increases [124]. Non-ionic surfactants, particularly polyethoxylated molecules, have the opposite behavior. KT is also known as the critical micelle temperature as it is connected to the critical micelle concentration (CMC) of the surfactant, as illustrated in Figure 13 [125,126]. The mechanism by which the bitumen droplets dispersion occurs is the incorporation of the bitumen molecules into the micelle. Therefore, temperature is then an important parameter in the formulation.

When it comes to the bitumen, higher temperature favors the development of smaller bitumen droplets. In this regard, studies indicate that a maximum bitumen viscosity of 200 mPa s is required for effective emulsification. This viscosity value cannot, in some cases, be achieved at manufacturing temperatures below 100 °C, particularly when using modified bitumen [127,128,129], as the viscosity of modified bitumen is often greater than that of untreated bitumen. Nevertheless, Gingras et al. noticed that, in some extreme instances, droplet size might be increased as a result of a temperature increase [108]. This can be explained by surfactant thermal fragility (thermal breaking at extremely high temperatures) or by the development of formulation as temperature rises (HLB declines, HLD approaches zero, and the system approaches optimal formulation) [130]. This can cause an immediate coalescence of a droplet. Keep in mind that the surfactant’s ability to keep the droplets repelling each other is not only required in the emulsification process but also will be required at storage.

The temperature of the water phase and the temperature of the bitumen phase [132] can be used to determine the emulsification temperature using the equation below:

(1) $$Tw\;=\;Te\;+\;\left(Te\;-\;Tb\right)\times \left(\;\frac{Cpb}{Cpw}\right)\times \;\left(\;\frac{b}{w}\right)$$

where:

*T_e_* = Temperature of emulsion °C;

$T_{b}\;$= Temperature of bitumen °C;

$C_{pb}$ = Heat capacity of bitumen;

$C{\;}_{pb}$ = 1.90 kJ/°C/kg;

$C_{pw}$= Heat capacity of water phase;

$C_{pw}$ = 4.18 kJ/°C/kg;

*w* = % of water phase;

*b* = % of bitumen phase;

$T_{w\;}$= Temperature of water phase °C.

In addition, Baumgardner [98] proposed an empirical equation based on soap and bitumen percentages and temperatures. The emulsification temperature can be empirically calculated using the following equation [98].

(2) $$ET=\frac{\left(Bw\times BT\times 0.5\right)+\left(SW\times ST\right)\;}{\left(Bw\times 0.5\right)+SW}$$

where:

$E_{T}$ = Emulsion temperature (°C);

$B_{w}$ = Bitumen weight (%);

$B_{T}$ = Bitumen Temperature (°C);

$S_{W}$ = Soap solution weight (%);

$S_{T}$ = Soap solution temperature (°C).

Both earlier equations do not take into account the emulsification time or the energy induced by tearing or milling the bitumen, which indeed causes a temperature rise as a function of emulsification time. Therefore, these parameters should be accounted for within the emulsification temperature estimation in order to obtain the required emulsion properties.

## 6. Bitumen Emulsion Formulation-Related Parameters

Formulators can change the final emulsion product by adjusting various formulation parameters. Among the other variables listed in Figure 7, they can change the surfactant, surfactant dosage, pH, water characteristics, emulsification temperatures, milling speed, emulsification time, and stator rotor gap. The manufacturer, on the other hand, must understand how changing the formulation of the bitumen emulsion affects its material properties and performance. Several studies have been carried out to determine how changing the formulation parameters of a bitumen emulsion affects its performance and material properties. As previously stated, the type of surfactant used in an emulsion has a significant impact on the charge and breaking speed of an emulsion. Surfactants have a lipophilic tail and a hydrophilic head group. Different combinations of tails and heads result in different charges and properties for the final emulsion product. The amount of surfactant in a soap solution also influences the properties and performance of emulsions [5]. Pang et al. [133] studied the influence of surfactant dosage on emulsion residue rheological performance. They discovered that increasing the surfactant content increases emulsion viscosity and modulus at the same temperature and frequency. It was also discovered that a higher surfactant content increased resistance to bitumen emulsion mixture deformation [133]. Miljkovi et al. explored the effect of cationic surfactant dosage on the cement hydration as well as the overall mechanical properties of bitumen emulsion on the mortar scale. The surfactant exerted a significant influence on water binding, cement hydration kinetics, and emulsion rheology characteristics. According to the findings of this study, surfactant dosage may be useful in understanding the mechanical performance of cold recycled mixtures [134]. In addition, Ouyang et al. [101] studied the surfactant dosage as well as other formulation parameters. They also investigated how the surfactant dosage affects viscosity and an emulsion’s ability to penetrate an aggregate base for a prime coat application. It was concluded that emulsions with a high surfactant content were preferred for use in prime coats due to their ability to effectively penetrate into the aggregate base. The pH of the aqueous phase has also been a major focus of study [101]. Xiao and Jiang [135] identified the effect of pH values on the physical properties of the resulting emulsion, such as sieve amount, viscosity, residue ductility, residue penetration, and residue softening point, as well as the emulsion’s storage stability. While each surfactant has a different optimum pH value, they concluded that adjusting the pH of the aqueous phase would have a significant impact on the emulsion’s residual amount on the sieve, viscosity, storage stability, and ductility of the residue. It was ascertained that the pH has a direct influence on the surfactant’s ability to disperse. This, in turn, affected the bitumen’s ability to emulsify and remain stable [135]. Cui and Pang [136] came to an agreement with Xiao and Jiang’s conclusion that optimizing the pH of the soap solution results in improved emulsion stability and performance. Nevertheless, they concluded that a high pH value resulted in lower interfacial tension, making bitumen emulsification easier [136]. In the presence of different electrolytes, Boucard et al. [137] investigated the performance of oil/water emulsions formulated with various oils with cationic surfactant quaternary ammonium salt. They discovered that the addition of an electrolyte promotes flocculation in all emulsions, with NaOH being the electrolyte that most promotes coalescence regardless of whether the dispersed phase is bitumen or silicone oil [137].

Water quality and purity also have an impact on the emulsion manufacturing process. Baumgardner [98] investigated the occurrence of mineral contaminants in water when employed in bitumen emulsions. To reduce mineral contaminants, an ion exchange must take place, with sodium being added to replace the magnesium and calcium ions frequently found in water. If these are not undertaken, the surfactant will lose its emulsifying characteristics, owing to undesired chemical interactions with magnesium and calcium [98]. Using molecular dynamic (MD) simulations, Kong et al. [138] studied the mass transfer mechanism of the anionic surfactant sodium dodecyl benzene sulfonate (SDBS), and its four isomers on the solid surface of calcium carbonate, and the resulting main chemical component of its aggregates. It was found that the SDBS and its isomers could be adsorbed on the calcium carbonate surface in a very short period of time and could subsequently form an aggregate structure throughout the mass transfer process. During this stage, there was no evidence of Na ion aggregation in the surfactant’s polar head [138].

Ziari et al. [139] assessed the effects of several manufacturing parameters such as milling variables, post-blending, and soap pre-batching, as well as the surfactant type on the properties of various modified bitumen emulsions with Styrene–Butadiene Rubber (SBR) latex. The results revealed that each kind of surfactant behaved differently in each process of polymer modification. Furthermore, the surfactant type and production technique impacted the mechanical characteristics of the mix, such as permanent deformation performance at high temperatures, fatigue cracking performance at intermediate temperatures, and other mechanical properties [139]. Table 2 summarizes the main results of the literature.

## 7. Bitumen Emulsion Formulation Tools

The bitumen emulsions are manufactured according to pre-established procedures in industrial applications. Therefore, specific formulation tools/theories have been presented in the literature. The next sections provide a brief overview of the most essential formulation tools, including the Winsor Ratio (R) theory, the Hydrophilic-Lipophilic Balance (HLB), and the Hydrophilic-Lipophilic Deviation (HLD) approach.

### 7.1. The Hydrophilic–Lipophilic Balance (HLB) Concept

In the last few decades, emulsion formulation (particularly bitumen emulsion) has been investigated but not fully understood. Griffin introduced the hydrophilic-lipophilic balance (HLB) in 1949, which assigns a surfactant an HLB value that specifies the type of emulsion that the surfactant would make. The selection of various surfactants in the emulsification process of bitumen emulsion is commonly still done empirically. Griffin’s HLB number [140] is a semi-empirical scale for choosing surfactants. Since then, numerous researchers have sought to establish a quick and reliable procedure for determining the HLB of every new surfactant as a measure of surfactant polarity [141]. The HLB criteria assess whether a surfactant is lipophilic or hydrophilic [142,143,144,145].

Much research has sought to link HLB to various features of surfactant molecules and to improve measuring methods for this parameter [11,146,147,148]. HLB values of surfactants were first determined through a time-consuming and arduous measurement of emulsion stability [149], particularly for cationic emulsions, for which there is limited information regarding HLB values in the literature. The HLB scale is based on the surfactant molecule’s relative fraction of hydrophilic to lipophilic (hydrophobic) groups (s). The HLB is a value ranging from 0 to 20 that reflects how easily a surfactant dissolves in oil or water. An HLB equal to 0 corresponds to an entirely hydrophobic (lipophilic) molecule, while a value of 20 corresponds to a molecule consisting entirely of hydrophilic components. The hydrophobic chain of an O/W emulsion droplet (such as bitumen emulsion) is in the oil phase, while the hydrophilic head group is in the aqueous phase.

The hydrophilic group(s) dwell in the water droplet of a W/O emulsion droplet, whereas the lipophilic groups dwell in the hydrocarbon phase [150]. The HLB number is determined by the type of oil. Surfactant HLB values are connected to various other characteristics such as CMC, solubility parameter (δ), and potential to cause emulsion inversion. The HLB value for non-ionic ethoxylated surfactants may be calculated using the following equation (known as Griffin’s equation) [151,152,153]:

(3) $${\mathrm{HLB}}_{\mathrm{Griffin}}=\frac{1}{5}(\frac{M_{H}}{M_{T}})\times 100$$

where:

M_H_ = The molecular mass of the hydrophilic part of the surfactant molecule;

M_T_ = The total molecular mass of the surfactant molecule. 

The HLB values, on the other hand, may be derived from their chemical formulas based on group numbers, as illustrated in Equation (4) based on Davies [152]:

(4) $${\mathrm{HLB}}_{\mathrm{Davies}}=7+\sum \left(\mathrm{hydrophilic}\;\mathrm{group}\;\mathrm{numbers}\right)\;-\sum \left(\mathrm{lipophilic}\;\mathrm{group}\;\mathrm{numbers}\right)$$

The following equation can be used to calculate the HLB value in the case of a surfactant having *n*-CH_2_^−^ groups:

(5) $${\mathrm{HLB}}_{\mathrm{Davies}}=7+\sum \left(\mathrm{hydrophilic}\;\mathrm{group}\;\mathrm{numbers}\right)-n\left({\mathrm{group}\;\mathrm{number}\;\mathrm{per}\;\mathrm{CH}}_{2}\mathrm{group}\right)$$

Based on the HLB method, Al-Sabagh was able to study the formulation of non-ionic bitumen emulsion using non-ionic surfactants (HLB range from 4 to 17.6) aiming to find a relationship between the HLB value and the stability of bitumen emulsion. In his study, bitumen emulsions were formulated with individual and some mixtures of surfactants. The highest stability was found to be in the HLB range of 10–13, with 70% bitumen (dispersed phase). The rheological results revealed that increasing the carbon numbers in the alkyl chain length increases the viscosity and stability of bitumen emulsions, hence slowing the rate of coalescence. However, this method has its downfalls, as it poorly accounts for temperature, salinity, and the nature of the oil, and it is also applicable primarily to non-ionic surfactants [154].

### 7.2. Winsor’s R-Ratio

Winsor [155] proposed a theoretical approach whereby the emulsion formulations might be assigned to a specific variable. This variable refers to the interaction between energies of adsorbed surfactant molecules with the oil and water in the system. It was demonstrated that the characteristics of an equilibrium system were strongly connected to a specific mix of interactions between surfactant, water, and oil. This interaction combination was denoted by the Winsor ratio (R) [155,156,157].

Winsor’s R-ratio [155] is a helpful way of understanding the phase behavior of surfactant–oil-water systems. It is stated as follows:

(6) $$R=\frac{A_{\mathrm{CO}}}{A_{\mathrm{CW}}}$$

where:

A_CO_: represents the interactions of the surfactant adsorbed at the interface with the oil;

A_CW:_ represents the interactions of the surfactant adsorbed at the interface with the water molecules.

The R value interpretation is based on the interaction between surfactant (S), oil (O), and water (W). In this regard, the emulsion is classified as Type I when R < 1, which means the W–S interaction is greater than the O–S interaction, while, when R > 1, the emulsion is considered Type II as the O–S interaction is greater than the W–S interaction. The system reaches a balance state when W–S and O–S interactions are balanced (optimum formulation), and therefore the emulsion is classified as Type III.

The interaction between emulsion components (SOW) varies when one parameter is changed; for example, raising the salinity reduces the interaction between the surfactant and the water, which leads to a reduced ACW. Consequently, the R-ratio would rise, and the emulsion system will likely migrate from Type I to Type III. In other words, augmenting the water salinity will raise the hydrophobicity of the surfactant system and intensify the interaction between the surfactant and the oil, raising the R-ratio. This means that greater hydrophilicity surfactants need larger salinity to create appropriate emulsions [157]. Nevertheless, unlike the HLB approach, this method’s R ratio could not even be determined numerically, making it impossible to utilize for practical emulsion formulations.

### 7.3. The Phase Inversion Temperature (PIT) Concept

Shinoda and Saito [158,159] are the first to introduce the PIT approach, which makes use of the unique ability of non-ionic surfactants. Non-ionic surfactants are hydrophilic at low temperatures due to the high hydration of the polar head group, which tends to be more soluble in water [160]. As the temperature increases, the polar head group dehydrates, causing it to become lipophilic. The amphiphilic nature of the surfactant is altered to a lipophilic activity, and the surfactant’s solubility in water diminishes. In the oil phase, the surfactant becomes more soluble than in the aqueous phase. Nevertheless, while shifting from a hydrophilic to a lipophilic nature, it reaches a temperature known as PIT, or the hydrophilic-lipophilic balance (HLB), at which neither lipophilicity nor hydrophilicity is present [161]. At this stage, the surfactant’s solubility in the oil and aqueous phases is about equal, with extremely low interfacial tensions [162,163,164].

The PIT method uses ultra-low interfacial tensions at the PIT or HLB temperature to facilitate obtaining very small droplet sizes. Nonetheless, it has been demonstrated that the emulsions are extremely unstable, with a very quick coalescence rate. As a result, Sherman and colleagues proposed using the PIT approach as a fast way to test emulsion stability, but this is preferably for short-term stability assessment, as this approach was created upon a relatively small number of surfactants and oils [165,166,167].

### 7.4. The Cohesive Energy Ratio (CER) Concept

Beerbower and colleagues [168] presented the Cohesive Energy Ratio (CER) method, trying to achieve a design notion that incorporates both the HLB values and Winsor’s R theoretical concept. From a conceptual standpoint, it was quite related to Winsor’s R, however, the ratio is between the surfactant layer’s adhesion energy to the oil phase and the surfactant layer’s adhesion energy to the water phase. The cohesion energy between molecules in a pure component system is calculated as follows:

(7) $$\delta^{2}=\frac{∆H_{Vap}}{\nu_{L}}$$

where the enthalpy of vaporization and the molar volume in the liquid state are denoted by $\;∆H_{Vap}$, $\nu_{L}$, respectively, and both are quantifiable numbers. δ  denotes the solubility parameter, which is directly related to the intermolecular cohesion forces. In the case of a mixed system, the adhesion forces between the two types of molecules are calculated using London’s geometric mean relationship, as illustrated below.

(8) $$\delta_{\left\{AA\right\}}^{2}=\frac{∆H_{AVap}}{\nu \cdot A_{L}}$$

(9) $$\delta_{\left\{BB\right\}}^{2}=\frac{∆H_{BVap}}{\nu \cdot B_{L}}$$

(10) $$\delta_{\left\{AB\right\}}^{2}=\delta_{\left\{AA\right\}}^{2}\delta_{\left\{BB\right\}}^{2}$$

Hundreds of compounds’ solubility parameters have been measured and tabulated, and they are widely used in conjunction with the standard solution model. This can be applied to measure the activity component in a mixture and in an individual into two phases. The adhesion between the surfactant and the oil phase was calculated by computing the $\delta_{\left\{AB\right\}}^{2}$ term, for which *A* represents the oil phase and *B* represents the surfactant’s lipophilic component, which was assumed to be equivalent to that of a hydrocarbon with the equivalent chain length [169]. The determination of the adhesion energy on the waterside of the contact is challenging due to a lack of experimental data for the hydrophilic group of specific surfactants. Thus, the cohesive energy ratio’s final result is erroneous.

### 7.5. Hydrophilic–Lipophilic Deviation (HLD)

**Figure 14:**
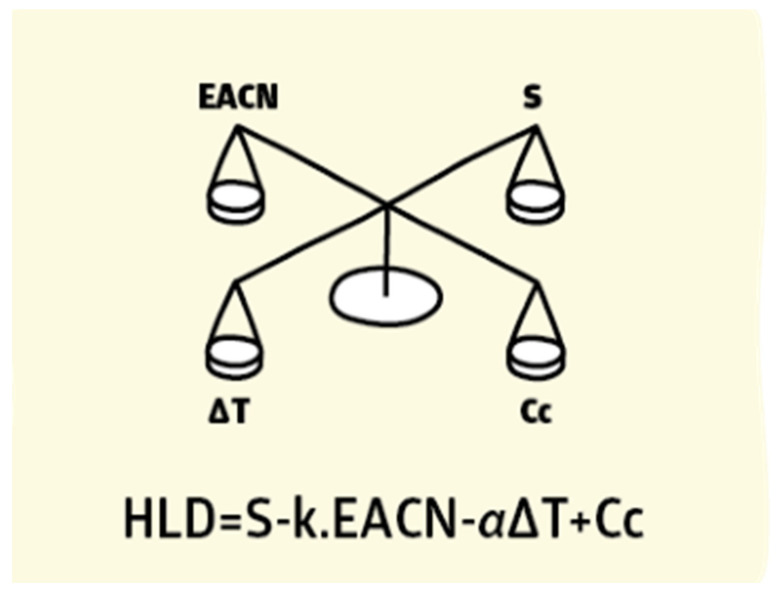
HLD as a balancing of system conditions [182].

**Figure 15:**
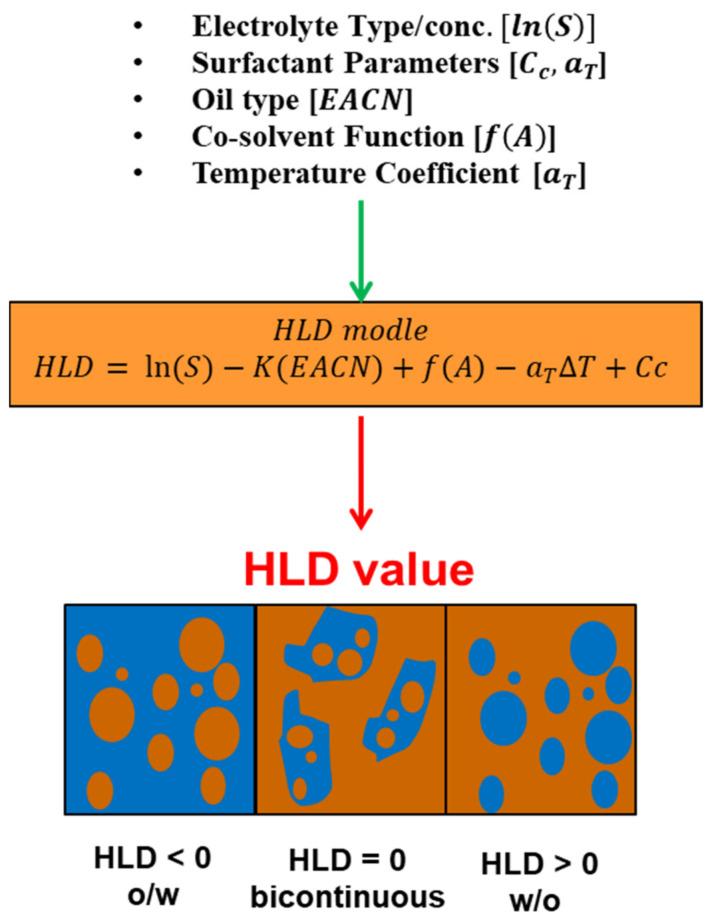
Inputs and outputs of HLD equation.

Due to the drawbacks of the HLB concept and the Winsor R ratio in calculating the optimum surfactant formulation processes [170,171,172,173,174,175], researchers [93,176,177,178] proposed a relatively newer approach that has gained significant support: the hydrophilic-lipophilic deviation (HLD). It is an improvement on the HLB system because it includes terms that account for the salinity, temperature, pressure, oil, and alcohol, as well as the surfactant’s hydrophobicity [157,179,180]. HLD is essentially a balance, as shown in Figure 14, and is expressed semi-empirically in Equation (11), between the salinity of the system, the Equivalent Alkane Carbon Number (EACN), the temperature, and the Characteristic Curvature (Cc) of the surfactant. When these terms are balanced (optimum formulation), the HLD is zero and the surfactant interacts with the water and oil phases equally [181], and thus the Type III microemulsions form, or Type IV if using an excessive surfactant, which gives the largest reduction of interfacial tension (ultra-low surface tension) and the largest increase in solubility. Due to the drawbacks of the HLB approach and the Winsor R ratio in determining the surfactant formulation procedures [170,171,172,173,174,175], researchers [93,176,177,178] developed a comparatively recent technique, the hydrophilic-lipophilic deviation, which has garnered substantial acceptance (HLD). It enhances the HLB system by including variables for salinity, temperature, pressure, oil, and alcohol, as well as the hydrophobicity of the surfactant [157,179,180]. As demonstrated in Figure 14, HLD is simply a balance between the salinity of the system, the Equivalent Alkane Carbon Number (EACN), the temperature, and the Characteristic Curvature (Cc) of the surfactant. When these terms are balanced (optimal formulation), the HLD is zero, the surfactant interacts equally with the water and oil phases [181], and Type III microemulsions form, or Type IV if excessive surfactant is used, which results in the greatest reduction of interfacial tension (ultra-low surface tension) and the greatest increase in solubility. Emulsions may be formed and identified based on HLD results, as seen in Figure 15. When the HLD value is less than zero, the emulsion is oil in water (O/W); when it is more than zero, it is water in oil (W/O), and zero refers to the ideal formulation (unstable emulsions tend to break extremely fast).

The HLD equation’s characteristic curvature term is beneficial in defining the hydrophilicity and lipophilicity characteristics of a wide range of surfactants and additives and the surfactant mixture [183]. The HLD-based emulsion formulation has been investigated in a variety of research areas, including enhanced oil recovery and detergent cleaning, drug delivery, and hard surface cleaning [172,184]. However, there is nearly no research on bitumen emulsion formulation published.

(11) $$HLD=\;\ln\left(S\right)-K\left(EACN\right)+f\left(A\right)-a_{T}∆T+Cc$$

where $S^{*}$ is the optimum salinity (g NaCl/100 mL of aqueous phase), K is the slope of the logarithm of the optimum salinity as a function of *EACN*, *EACN* is an equivalent alkane carbon number of the oil, *f*(*A*) is a function of alcohol content, which is zero with no alcohol involved, Cc is a characteristic curvature of the surfactant, $a_{T}$ is a temperature coefficient of optimum salinity, and *T* is the temperature in °C [185].

#### 7.5.1. Terms Characterizing for HLD Equation

The term $ln\left(S\right)$ is responsible for the effect of ionic strength on film curvature. It is presented as a function of the aqueous phase salinity (*S*) expressed as equivalent grams of NaCl or HCL (depending on surfactant charge) per 100 mL [186].

**Figure 16:**
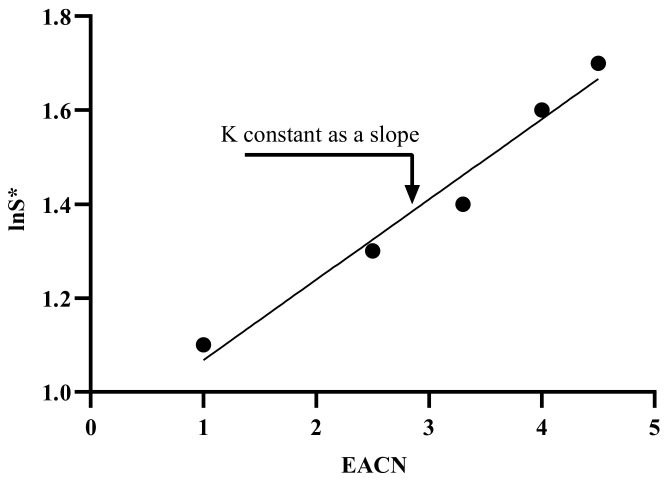
Determining K constants as the slope [172].

The abbreviation $a_{T}$ΔT describes the influence of temperature variations regarding the reference temperature (25 °C), where $a_{T}$ is slightly positive for ionic surfactants (0.01–0.02 for cationic surfactants) and is negative for ethoxylated surfactants [187]; ΔT is the temperature difference between the actual and reference temperatures [188]. In order to obtain the $a_{T}$ value, a graphical relationship between optimal salinity and temperature for a particular surfactant and oil can be used, and the arithmetic slope represents the $a_{T}$. In general, the slope values for each anionic, cationic, and non-ionic are often identical and presumed constant, 0.01, 0.02, and 0.06, respectively [189]. K is affected by the surfactant’s head group [190,191]. Figure 16 depicts the slope of the observed linear correlation between the logarithm of optimal salinity and the EACN. When all other formulation factors are held constant, the lipophilic interactions between the surfactant’s hydrophobic tail and the oil are reflected in K and EACN. K is often dependent on the surfactant family, with values ranging from 0.16 for alkylbenzene sulfonates to 0.10 for fatty acid sodium soaps [192].

Cc is a surfactant characteristic variable that defines the degree to which a surfactant is more hydrophilic or hydrophobic. A hydrophilic surfactant has a negative Cc value, while a hydrophobic surfactant has a positive Cc value. In other words, the Cc parameter describes the surfactant’s tendency to cause the interface to curve away from the aqueous phase (hydrophilic surfactant) or away from the oil phase (hydrophobic surfactant). Higher hydrophobic surfactants have a higher positive Cc and promote a positive HLD [190,193].

The primary method of determining Cc is scanning. A scan for an unknown Cc would involve a series of test tubes, the surfactant in question, an oil with known EACN, a salt, and, depending on the scan, an additional oil with known EACN or surfactant with known Cc. The EACN (equivalent alkane carbon number) is often used to represent the influence of the oil phase and refers to the number of carbon atoms in the case of the linear alkanes. EACN can be established and calculated empirically in the case of oil other than *n*-alkanes (2.5 for bitumen) [194]. A K constant is used in the model to scale the EACN for the reason of obtaining a reasonable sum with the salinity function.

#### 7.5.2. HLD Emulsion Formulation Diagram

**Figure 17:**
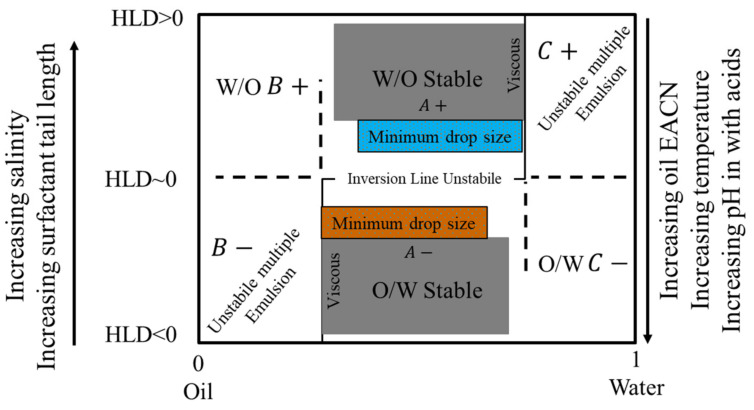
Classical formulation-composition diagram (HLD-WOR) showing the inversion line and emulsion types and the basic properties (stability, drop size, and viscosity) adapted from [195].

HLD has been used frequently to describe regions where emulsions exhibit a given behavior, where they are stable, and where they would invert. Salager et al. [195] graphically represented the HLD values with the emulsion type and inversion line. It is the most important map for describing how to determine an emulsion characteristic needed for a certain application. When the formulation is modified or tweaked in relation to the bitumen/aqueous phase composition, a plethora of emulsion types and attributes become available [185]. In Figure 17, the emulsion inversion is depicted by the horizontal line at HLD 0 with the WIII phase behavior in the WOR middle region, i.e., when one of the phases (bitumen/aqueous phase) is less than 70–75%. The normal emulsion (O/W or W/O) may be obtained from the diagram where the HLD formulation is in the region of A+ and A−. The extreme zones are known as B+ (or C−), which can be thought of as the continuation of the A+ (or A−) zone with a high dilution. The B− and C+ zones, on the other hand, are considered to be abnormal because they have multiple emulsions, with droplets in drops creating the internal emulsion and drops in a continuous phase producing the exterior emulsion. The same graphic may be used to predict emulsion fundamental features such as smaller droplets or higher viscosity, as well as stable emulsions [196]. At HLD 0, there is a very unstable emulsion zone at the horizontal center section of the inversion line, which tends to break very fast immediately as soon as it comes into touch with the destabilizer agent (i.e., cement).

## 8. Conclusions

This study summarizes the previous studies aiming to expand the knowledge of bitumen emulsion manufacturing, its fundamental components, and the formulation tools needed to further maximize its applications in practice. The study’s conclusions are summarized below:

- The physicochemical formulation of the surfactant-bitumen-water system is discussed extensively.
- Bitumen emulsification processes were identified as a colloid mill and the HIPR method. However, the colloid mill method could not be used for stiffer bitumen, while the HIPR approach may produce a concentrated emulsion with an average droplet size of 1 μm.
- In the formulation process, several parameters, such as materials fractions, mill speed, pH of soap phase, and using the HIPR method, can have a significant impact on specific emulsion properties such as droplet size distribution.
- Bitumen aggregate affinity can be improved by optimizing bitumen emulsion production parameters. For example, adding some salt will increase the viscosity, which in turn affects the adhesion work between the aggregate and the bitumen.
- When utilizing fatty acids or amine ionic surfactants, formulation acidity dependence is a critical parameter to consider (using HCl or NaCl for soap preparation).
- This comprehensive review gathered scientific literature to help in producing bitumen emulsion with the required properties for specific applications using scientific and pre-established approaches. Various formulation tools were presented and reviewed. HLD could be used to formulate and analyze the bitumen emulsification process, as well as anticipate the resulting emulsion characteristics, which can offer an insight into pavement performance to the mix designer. This can be helpful to use the ultimate potential of bitumen emulsion.
- Although some theoretical formulation tools have been described in this paper, experimental work is required to initiate the use of these theories such as salinity scans, perturbation experiments, etc.

There is still an edge for more inventive research since there is no doubt that the commonly employed random trial and error processes for producing bitumen emulsion are destined to fail due to the vast number of variables. One of the goals of this study was to persuade the reader that a large number of know-hows does exit in an easy-organized review, which could be useful to carry out bitumen emulsion formulation engineering tasks for future work.

## Figures and Tables

**Table 1:** Cationic emulsion abbreviated description adapted from [27].

Symbol	Description	Standard
C	Cationic bitumen emulsion	EN 1430
Two digit number	Bitumen content as a% by mass	EN 1428 EN 1431
	Type of binder	
B	Bitumen grade	EN 12591
P	Addition of polymers	EN 14023
F	Addition of more than 3% by mass of flux	
2 to 10	Breaking value	EN 13808

**Table 2:** Summary of studies on bitumen emulsion formulation parameters.

Study	Variable	Conclusion
Pang et al. [133]	Surfactant content	Upsurging the surfactant content increases the viscosity of the emulsion.
Miljković et al. [134]	Cationic surfactant content	The surfactant content affects the cement hydration kinetics, emulsion rheology, and water binding, which are linked to mechanical performance cold mix.
Ouyang et al. [101]	Surfactant content	Higher surfactant content in prime coat emulsion resulted in higher aggregate base interlocking.
Xiao and Jiang [135]	The pH of the aqueous phase	pH values are correlated with surfactants and affect the resulting final emulsion properties.
Cui and Pang [136]	The pH of the aqueous phase	The interfacial tension property is dependent on the pH value of the aqueous phase.
Boucard et al. [137]	Electrolyte type	The addition of an electrolyte promotes flocculation, while the electrolyte NaOH promotes coalescence regardless of the dispersed phase (bitumen or silicone oil).
Baumgardner [98]	Ionic exchange	Ion exchange should take place, enabling the emulsion to retain its properties.
Baumgardner [98]	Colloid mill parameters	Input and output temperatures as well as the milling mechanical variables all have a significant impact on the properties of the final bitumen emulsions.
Kong et al. [138]	Anionic surfactant structure	During the mass transfer process, the SDBS and its isomers were adsorbed on the calcium carbonate surface and produced an aggregate structure. Na ions exhibited no evident aggregation behavior in the surfactant’s polar head during this phase.
Ziari et al. [139]	Surfactant type and emulsification method	The surfactant type and manufacturing technique seemed to have an impact on the mechanical characteristics of the mix, such as permanent deformation performance at elevated temperatures, fatigue cracking performance at intermediate temperatures, and some other mechanical properties.
Tan et al. [87]	Surfactant type and its content	The surfactant has a considerable retarding impact on cement hydration, which is related to the surfactant kinds and doses.

## Data Availability

No new data were created or analyzed in this study. Data sharing is not applicable to this article.
